# Normal tissue doses from MV image‐guided radiation therapy (IGRT) using orthogonal MV and MV‐CBCT

**DOI:** 10.1002/acm2.12276

**Published:** 2018-03-03

**Authors:** Yuting Li, Tucker Netherton, Paige L. Nitsch, Peter A. Balter, Song Gao, Ann H. Klopp, Laurence E. Court

**Affiliations:** ^1^ Department of Radiation Physics Division of Radiation Oncology The University of Texas MD Anderson Cancer Center Houston TX USA; ^2^ Department of Radiation Oncology Ohio State University Medical Center Columbus OH USA; ^3^ The University of Texas Graduate School of Biomedical Sciences at Houston Houston TX USA

**Keywords:** halcyon, MV‐IGRT, normal tissue imaging dose

## Abstract

**Purpose:**

The aim of this study was to measure and compare the mega‐voltage imaging dose from the Halcyon medical linear accelerator (Varian Medical Systems) with measured imaging doses with the dose calculated by Eclipse treatment planning system.

**Methods:**

An anthropomorphic thorax phantom was imaged using all imaging techniques available with the Halcyon linac — MV cone‐beam computed tomography (MV‐CBCT) and orthogonal anterior‐posterior/lateral pairs (MV‐MV), both with high‐quality and low‐dose modes. In total, 54 imaging technique, isocenter position, and field size combinations were evaluated. The imaging doses delivered to 11 points in the phantom (in‐target and extra‐target) were measured using an ion chamber, and compared with the imaging doses calculated using Eclipse.

**Results:**

For high‐quality MV‐MV mode, the mean extra‐target doses delivered to the heart, left lung, right lung and spine were 1.18, 1.64, 0.80, and 1.11 cGy per fraction, respectively. The corresponding mean in‐target doses were 3.36, 3.72, 2.61, and 2.69 cGy per fraction, respectively. For MV‐MV technique, the extra‐target imaging dose had greater variation and dependency on imaging field size than did the in‐target dose. Compared to MV‐MV technique, the imaging dose from MV‐CBCT was less sensitive to the location of the organ relative to the treatment field. For high‐quality MV‐CBCT mode, the mean imaging doses to the heart, left lung, right lung, and spine were 8.45, 7.16, 7.19, and 6.51 cGy per fraction, respectively. For both MV‐MV and MV‐CBCT techniques, the low‐dose mode resulted in an imaging dose about half of that in high‐quality mode.

**Conclusion:**

The in‐target doses due to MV imaging using the Halcyon ranged from 0.59 to 9.75 cGy, depending on the choice of imaging technique. Extra‐target doses from MV‐MV technique ranged from 0 to 2.54 cGy. The MV imaging dose was accurately calculated by Eclipse, with maximum differences less than 0.5% of a typical treatment dose (assuming a 60 Gy prescription). Therefore, the cumulative imaging and treatment plan dose distribution can be expected to accurately reflect the actual dose.

## 1 INTRODUCTION

On‐line mega‐voltage (MV) imaging has been successfully used for daily image‐guided patient setup for many years, including orthogonal imaging, helical computed tomography (CT), and cone‐beam CT (CBCT).^1^, ^2^, ^3^, ^4^, ^5^ Since the early 2000s, however, there has been a significant increase in the use of kilo‐voltage imaging for in‐room patient setup, including orthogonal imaging, helical CT (CT‐on‐rails), and CBCT.^6^ Although kilo‐voltage imaging offers the advantages over MV imaging of lower patient dose and better soft‐tissue contrast, MV imaging has the potential advantages of reduced equipment costs and complexity, which can be expected to transfer to gains in reliability.

Varian Medical Systems (Palo Alto, CA) recently released Halcyon, a medical linear accelerator which uses daily MV imaging for patient setup.^7^ Since there's a concern that MV‐IGRT would introduce higher normal tissue dose compared with kV IGRT,^8^, ^9^ in this present study we performed extensive measurements of the imaging dose for all available imaging techniques on the Halcyon linac for tissues within and outside the treatment volume. We then compared the measured doses with those calculated by Eclipse, to verify that the Eclipse beam model, which is pre‐commissioned for Halcyon, could correctly predict the MV imaging dose and incorporate it into the treatment plan. This report adds practical data specific to the Halcyon, beyond what has been previously reported for other systems.^9^, ^10^, ^11^

## 2 MATERIALS AND METHOD

### 2.A Phantom selection

We used a heterogeneous anthropomorphic thorax phantom (0002LFC; CIRS, Norfolk, VA) with a breast phantom attached to the right chest for both ionization chamber measurements and TPS calculation of the imaging dose to normal tissue structures. This phantom contains ion chamber inserts within tissue equivalent media for the lungs, spine, mediastinum, and breast. The phantom was scanned using CT and the CT images were imported into the Eclipse TPS.

### 2.B Treatment device and available imaging modalities

The Halcyon has an enclosed, ring‐mounted gantry with an opposing electronic portal imaging device (EPID) and beam stopper. A 6 MV flattening filter free (FFF) beam is used for both treatment and imaging on this machine, and the daily imaging dose is incorporated into the treatment plan (in the Eclipse treatment planning system). The maximum field size (28 × 28 cm^2^) at the isocenter (1 m) is defined by a dual‐layer multileaf collimator (MLC) system without physical jaws or light field. Using the internal lasers, the patient is aligned to a virtual isocenter and then shifted into the bore. Subsequent treatment relies entirely on MV‐image guidance. The imaging dose is calculated by the treatment planning system (TPS) and incorporated into the final treatment plan.

**Figure 1:**
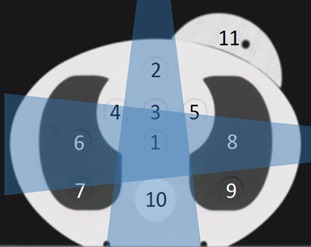
Anthropomorphic phantom (CIRS) with dose measurement points and isocenter locations identified. Point 11 is offset by 5.5 cm longitudinally from points 1–10. In this study, isocenter was located at point 1, 2, 6, 8, 10 and 12, which is in the center of the phantom with an 8 cm offset from the axial plane of points 1–10. The target region of MV‐MV is illustrated as dark blue (intersection) region. The remaining part is defined as extra‐target region.

Two imaging modalities are available on the Halcyon system: orthogonal anterior‐posterior/lateral pairs (MV‐MV) and MV‐CBCT, each with “Low‐Dose” and “High‐Quality” modes. MV‐MV has fixed gantry angles of 0° and 90° (Fig. 1) with total of two monitor units (MU) for low‐dose mode and 4 MU for high‐quality mode. The collimator angle is fixed at 0° during imaging. The imaging field size can be adjusted by changing leaf separation along the y‐axis to any number less than or equal to 28 cm and separation along the x‐axis to any even integer less or equal to 28 cm.

MV‐CBCT images are acquired via a continuous gantry rotation from 260° to 100°, with total of 5 MU for low‐dose mode and 10 MU for high‐quality mode. The collimator angle is fixed at 0° during imaging. For MV‐CBCT, the axial field‐of‐view (FOV) could not be adjusted because it is fixed at 28 cm on Halcyon, but the FOV longitudinal length can be changed from 2 cm to 28 cm in 2 cm increments.

### 2.C Imaging dose calculations

Using MV‐MV and MV‐CBCT in both low‐dose and high‐quality modes, we created 54 treatment plans in Eclipse (v15.5) with different imaging field sizes (Table 1) and isocenter locations (Fig. 1). The Fourier Transform Dose Calculation (FTDC) Algorithm is used for calculating imaging dose and is built within the Analytic Anisotropic Algorithm (AAA) dose calculation algorithm.^11^ The FTDC uses a convolution/superposition algorithm, that is, optimized for speed by simplifying AAA's three‐source dose model and uses a 5.0 mm calculation resolution. This dose calculation algorithm is pre‐loaded into the Eclipse planning system, and no changes by the user are possible. Imaging doses delivered to 11 points in the heart, lungs, spine, and breast were calculated in Eclipse and later compared with our corresponding measurements described below.

**Table 1:** Isocenter locations and field sizes where normal tissue imaging doses were measured using Halcyon and in Eclipse. The location numbers are referred to the points identified in Fig. 1

Measurements		
Isocenter location	Field size (cm^2^)	
	MV‐MV	MV‐CBCT
1	2 × 2, 6 × 6, 10 × 10, 14 × 14, 20 × 20, 28 × 28	2 × 28, 6 × 28, 10 × 28, 14 × 28, 20 × 28, 28 × 28
1, 2, 6, 8, 10, 12	28 × 28	28 × 28

### 2.D Imaging dose measurements

The phantom was placed in the treatment position, images were taken, and imaging doses were measured using a small volume ion chamber (CC04; IBA Dosimetry, Bartlett, TN) after cross‐calibration with a Farmer chamber (with Accredited Dosimetry Calibration Laboratory calibration). Imaging dose measurements were taken at 11 points (Fig. 1): Points 1–10 were in a single axial plane, and point 11 was offset in the longitudinal direction by 5.5 cm. All imaging was performed using the Halcyon system in clinical mode. Each measurement was repeated three times, and the average calculated.

To examine the effect of collimation during imaging upon normal tissue imaging doses, the isocenter was placed in the center of the phantom (point 1 in Fig. 1), and the imaging doses from various imaging field sizes were measured. To evaluate the effect of isocenter location on imaging dose, we used the maximum field size (28 × 28 cm^2^) and placed the isocenter at points 2, 6, 8, 10, and 12 (Table 1), where point 12 is in the center of the phantom with an 8 cm offset from the axial plane of points 1–10. For MV‐MV technique, imaging dose data were designated as “target‐region” if the point being evaluated was within the intersection between the AP and lateral imaging beams, shown as the dark blue region in Fig. 1. Data measured on all other points outside of this region were labeled “extra‐target region”.

For MV‐CBCT, we measured the imaging dose with various field sizes: 2 × 28, 6 × 28, 10 × 28, 14 × 28, 20 × 28 and 28 × 28 cm^2^ with the isocenter at point 1. We also examined the imaging dose with the isocenter at the same six isocenter locations used in the MV‐MV study with the MV‐CBCT field size fixed at its maximum (Table 1).

### 2.E Breast imaging dose

Since the arrangement of the orthogonal imaging pair (MV‐MV) can only be at 0° and 90°, we studied the imaging doses to the left and right breasts. Because of the Halcyon linac's bore dimension (1 m diameter), for large patients, placing the isocenter in the treated breast or chestwall was impossible. Thus, for these patients, the isocenter was placed in the ipsilateral lung to avoid collision with the bore. To simulate all possible isocenter locations for breast and chestwall treatments using the Halcyon, we evaluated the imaging doses of ipsilateral and contralateral breasts with the isocenter located in the target breast or ipsilateral lung at the maximum field size (28 × 28 cm^2^) for both MV‐MV and MV‐CBCT modalities. All the isocenter locations for breast imaging dose measurements were in the same axial plane.

## 3 RESULTS

### 3.A Normal tissue dose

**Figure 2:**
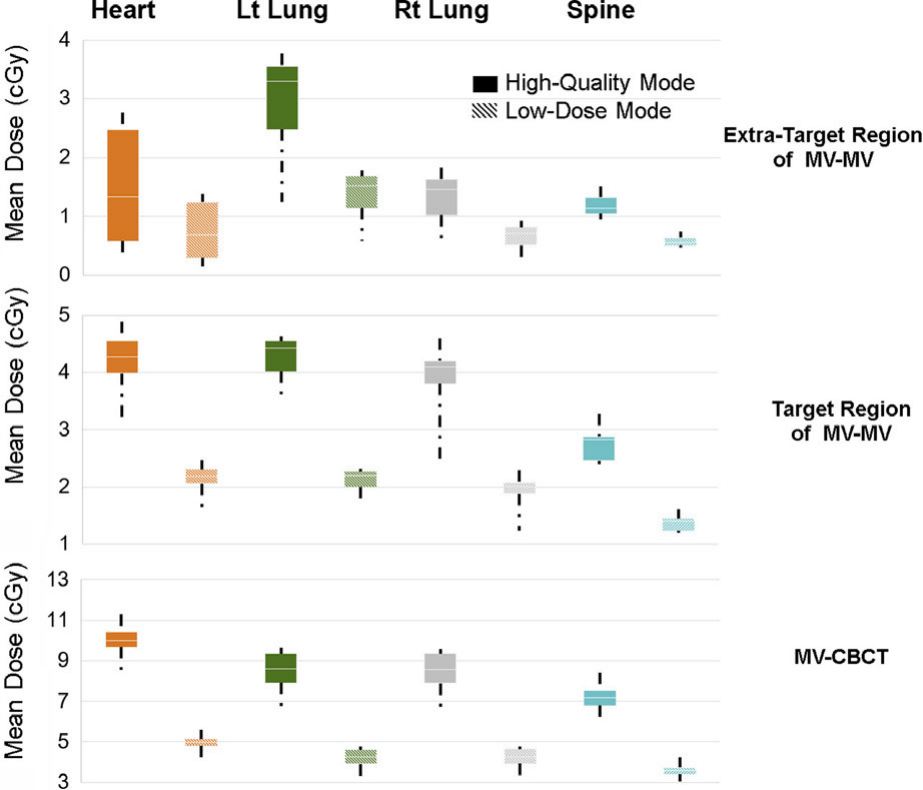
Normal tissue imaging dose of the extra‐target and target regions of MV‐MV and of MV‐CBCT techniques. Measured data of all measured field sizes and isocenter locations were included in the figure.

**Figure 3:**
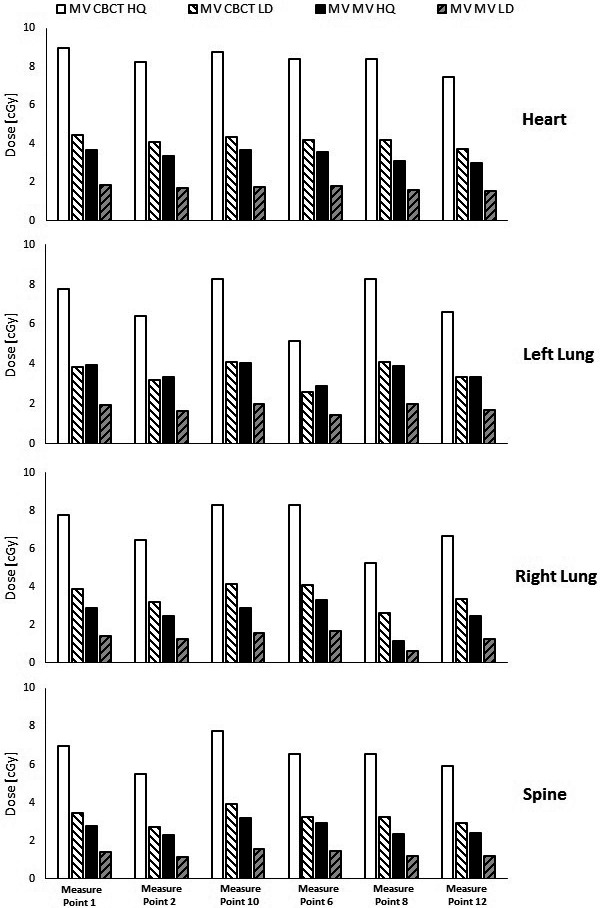
Variation in normal tissue imaging dose depending on isocenter location. HQ, high‐quality; LD, low‐dose.

Fig. 2 shows the imaging doses measured at points in the heart, left lung, right lung, and spine, averaged for all measured field sizes and isocenter locations. For MV‐MV, we categorized the tissue doses according to their locations relative to the target volume — extra‐target and target regions, as shown in Fig. 2. We found that for high‐quality MV‐MV mode, the mean extra‐target doses of heart, left lung, right lung and spine were 1.184, 1.644, 0,802 and 1.105 cGy per fraction, respectively. The corresponding in‐target doses were 3.358, 3.719, 2.612 and 2.686 cGy per fraction, respectively. Moreover, the imaging doses in the extra‐target regions from MV‐MV technique exhibited higher variation and dependency on imaging field size than did those in the in‐target regions. The imaging dose from MV‐CBCT was less sensitive to the location of the organ relative to the treatment field than MV‐MV technique. For high‐quality mode, the mean imaging doses delivered to the heart, left lung, right lung and spine were 8.448, 7.158, 7.190, and 6.512 cGy per fraction, respectively. For both MV‐MV and MV‐CBCT techniques, imaging doses to the lung were more sensitive to changes of isocenter location than those of other organs, with a maximum variation of 3.12 cGy in the left lung for high‐quality MV‐CBCT (Fig. 3). In both MV‐MV and MV‐CBCT cases, use of the low‐dose mode resulted in an imaging dose about half of that using the high‐quality mode.

### 3.B Breast dose

Table 2 shows the measured breast imaging doses using MV‐MV and MV‐CBCT in both low‐dose and high‐quality modes. Each data point is an average of measured values at two isocenter locations (in target breast and in ipsilateral lung). The first column in the Table 2 indicates the breast of interest where imaging dose measurements were taken, whereas the second column describes the position of the breast of interest relative to the actual treatment site. For MV‐MV imaging, the breast dose depended on both the treatment location and laterality. The doses for the high‐quality mode ranged from 0.8 cGy to 4.3 cGy. Because the lateral image is always taken with the gantry at 90°, the left breast is closest to the x ray source. For this reason, the lowest imaging dose was to the right contralateral breast when the left breast was the target. The highest imaging dose was to the left ipsilateral breast when the left breast was the target. In the cases with same side laterality, the left breast always received higher doses than did then right breast, because the orthogonal imaging fields for Halcyon are fixed at 0° and 90°. For high‐quality mode, when the breast of interest was the one being treated, the imaging dose to the left breast was about 0.9 cGy higher than the right breast. When the breast of interest was contralateral to the treated site, the left breast received a 1.9 cGy higher imaging dose than the right breast would. The imaging doses received by the breasts from CBCT were less sensitive to the treatment site location than in MV‐MV and were only a function of target laterality. In all cases, the imaging doses administered in low‐dose mode were approximately half of those from the high‐quality mode.

**Table 2:** Measured imaging doses per fraction to the breast with daily MV‐MV and MV‐CBCT imaging using the maximum field size of 28 × 28 cm^2^

Breast of interest	Relative position to treatment site	MV‐MV (cGy)		MV‐CBCT (cGy)	
		Low‐dose	High‐quality	Low‐dose	High‐quality
Left	Ipsilateral	2.15	4.32	4.82	9.71
Right		1.71	3.40	4.76	9.64
Left	Contralateral	1.35	2.76	2.85	5.77
Right		0.41	0.83	2.83	5.72

### 3.C Comparison of measurements and TPS calculations

**Figure 4:**
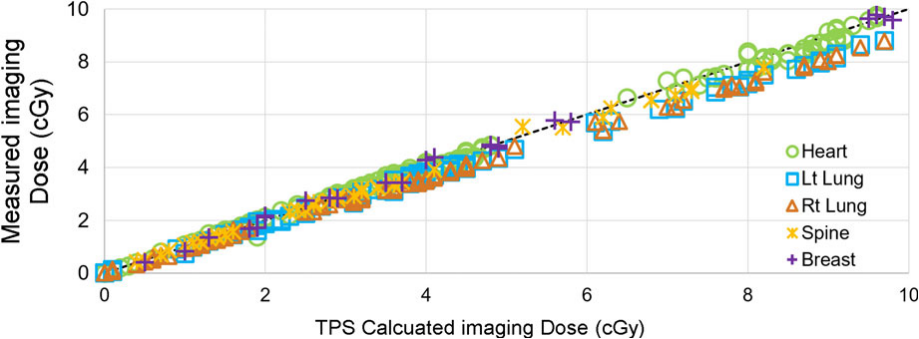
Comparison of measured and eclipse calculated normal tissue imaging doses.

The average differences between the Eclipse calculated and measured imaging doses had were −0.05, −0.35, −0.35, −0.09, and 0.01 cGy for the heart, left lung, right lung, spine, and breast, respectively. As shown in Fig. 4, the TPS tended to overestimate the imaging dose in nearly all cases, with the largest disagreement being in the lung (0.99 cGy).

## 4 DISCUSSION

MV imaging is fully integrated into the patient treatment workflow for the Halcyon medical linear accelerator. Therefore, it is crucial to understand the (a) impact of imaging doses to normal tissue, and (b) accuracy of MV imaging dose calculation in the treatment planning system. Therefore, we investigated this for all of the imaging techniques offered by Halcyon: MV‐MV and MV‐CBCT, both with high‐quality and low‐dose mode. The pre‐commissioned Eclipse paired with Halcyon exhibited good accuracy in calculating the imaging dose. Based on our imaging dose measurements, for treatment with 30 fractions, the maximum difference between the calculated and actual imaging dose would be about 30 cGy, or approximately 0.5% of the target dose (assuming a 60 Gy prescription).

Accurately including the imaging dose in the treatment plan is important for several reasons. It means that it is possible to perform sensible comparisons of plans treated with different treatment devices and different imaging approaches (e.g., comparison with devices with much lower imaging dose). It also means that clinical experience in terms of normal tissue dose constraints can be directly applied to the Halcyon treatment plans.

Although other researchers have evaluated MV imaging doses for other systems, we are the first to report imaging dose measurements with the Halcyon treatment delivery system. This information will be useful to users of this new device when introducing it into clinical use.

## 5 CONCLUSIONS

The in‐target doses due to MV imaging using the Halcyon ranged from 0.59 to 9.75 cGy, depending on the choice of imaging technique. Extra‐target doses from MV‐MV technique ranged from 0 to 2.54 cGy. The MV imaging dose was accurately calculated by Eclipse, with maximum differences less than 0.5% of a typical treatment dose (assuming a 60 Gy prescription). Therefore, the cumulative imaging and treatment plan dose distribution can be expected to accurately reflect the actual dose.

## CONFLICT OF INTEREST

This work was partially funded by Varian Medical Systems.
